# Supplementary material for: Higher order effects in organic LEDs with sub-bandgap turn-on
Source: Nat Commun. 2019 Jan 16;10:227. doi: 10.1038/s41467-018-08075-z (PMC6335427; doi:10.1038/s41467-018-08075-z)
Supplement: Supplementary file 1 — Supplementary Information [file 41467_2018_8075_MOESM1_ESM.pdf]

Higher Order Effects in Organic LEDs with Sub-bandgap Turn-on

Sebastian Engmann[1,2], Adam J. Barito[2], Emily G. Bittle[2], Noel C. Giebink[3], Lee J. Richter[4], David J. Gundlach[2]

[1]Theiss Research, 7411 Eads Ave. La Jolla, California 92037-5037

[2]Nanoscale Device Characterization Division, National Institute of Standards and Technology, 101 Bureau Drive, Gaithersburg, Maryland 20899

[3]Department of Electrical Engineering, The Pennsylvania State University, Electrical Engineering West, State College, Pennsylvania 16801

[4]Materials Science and Engineering Division, National Institute of Standards and Technology, 101 Bureau Drive, Gaithersburg, Maryland 20899

Correspondence and requests for materials should be addressed to D.J.G. (email: david.gundlach@nist.gov) or S.E (email: sebastian.engmann@theissresearch.org)

Supplementary Information

Supplementary Figures

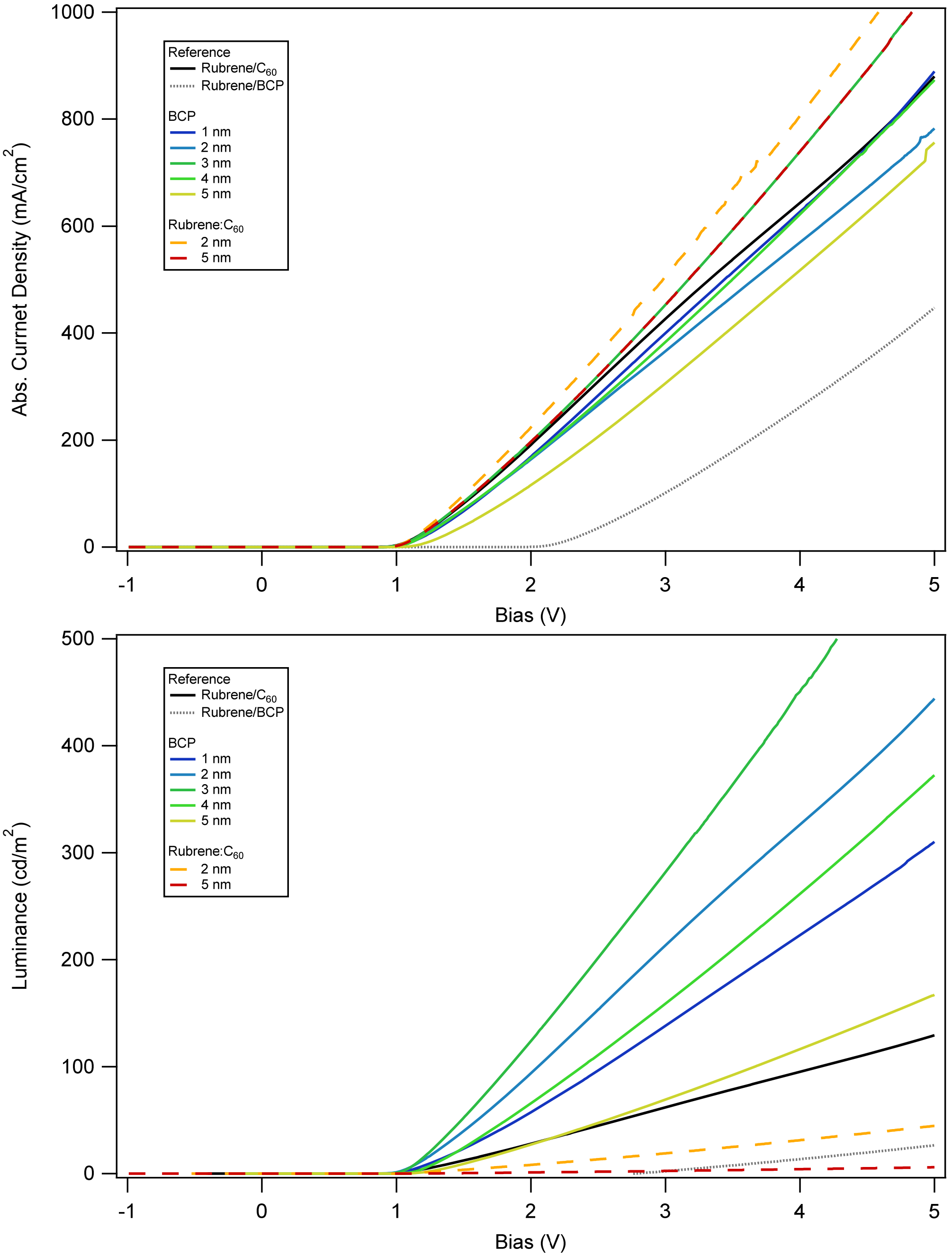

Supplementary Figure 1 – Current Density – Luminance – Voltage characteristics

J(*V*)-, *L*(*V*)- of rubrene/C_{60} OLEDs with BCP or Rubrene:C60 (1:1) interlayers inserted between the rubrene and C_{60} layers. Replot of Figure 2 on a linear scale.

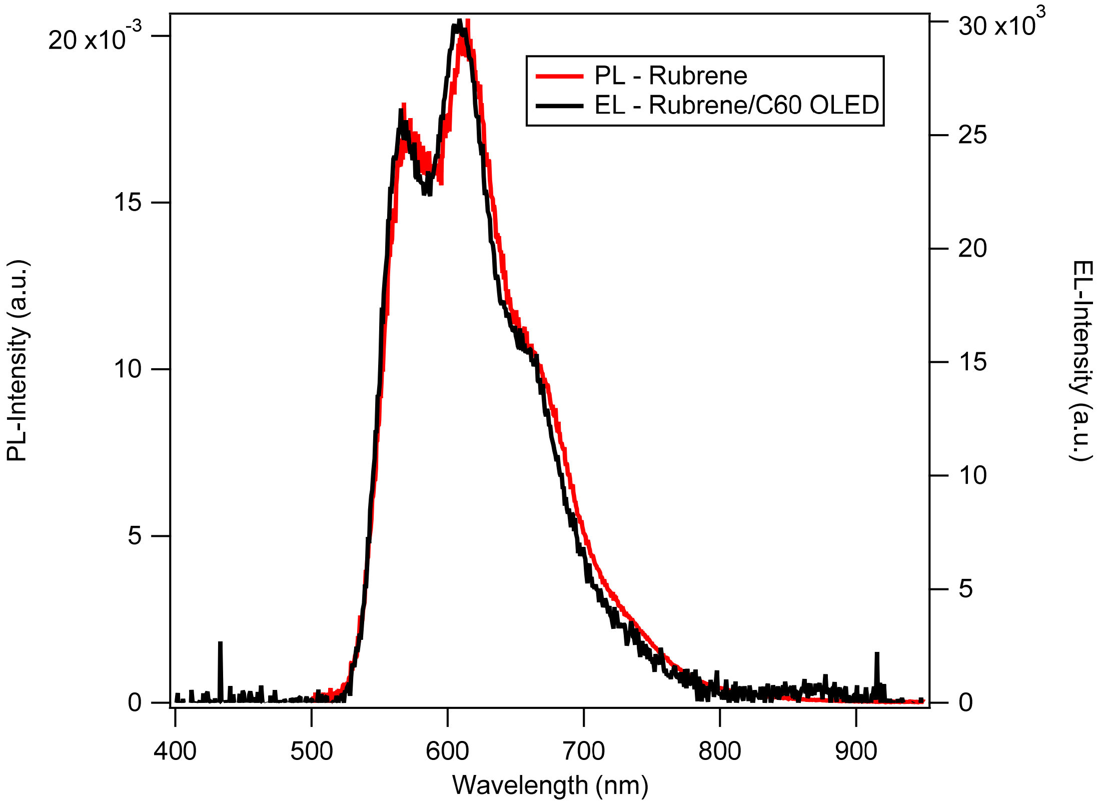

Supplementary Figure 2 – Rubrene photoluminescence spectra

Photoluminescence of a single rubrene layer in comparison with electroluminescence from a rubrene/C_{60} OLED.

Supplementary Note 1: Magneto-electroluminescence (MEL) measurements

In addition to the electrical characterization, we investigated the magneto-electroluminescence (MEL) of the devices with BCP interlayer. It has been discussed often that an Auger assisted up conversion process significantly differs from a TTA mechanism in its MEL-characteristics. TTA shows an increase in luminescence with small applied magnetic fields, before a steady decrease in the luminescence can be observed for large fields.[1-4] An Auger process should have a somewhat inverted dependence as singlet fission becomes dominant and increased fields lead to increased MEL.[5,6] The measured MEL-characteristics for our devices, shown in **Supplementary Figure 3**, agree well with those reported for other materials exhibiting a triplet-triplet annihilation mechanism,[1-4] luminescence increase for small fields (here <25 mT) and steady decrease (here 25 mT < B < 100 mT) thereafter. However, the relative change in MEL response is very small, between +1 % and -4 %, such that this result is not conclusive and might be due to small changes in recombination dynamics of free charges independent of TTA.

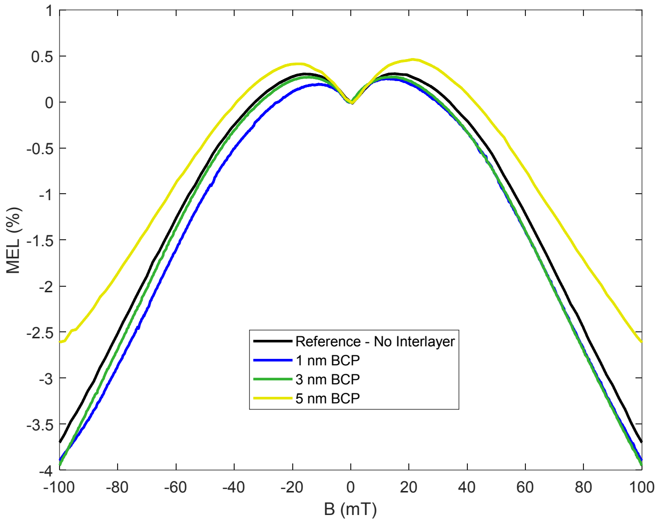

Supplementary Figure 3 – Magneto electroluminescence spectra of rubrene/C_{60} OLEDs

Magneto electroluminescence spectra of rubrene/C_{60} OLEDs for 0, 1, 3, and 5 nm BCP interlayer thicknesses at room temperature.

Supplementary Note 2: Considerations on Diode Ideality

Ideality Factor n and recombination current density J_{rec}:

$$J_{rec} = \int qRdx \propto \exp\left(\frac{qV}{nk_BT}\right) \tag{1}$$

Bimolecular recombination

$$R_{bi} \approx k_{bi}np \approx k_{bi}n_i^2 \exp\left(-\frac{E_g}{k_bT}\right)\exp\left(\frac{qV}{k_BT}\right) \tag{2}$$

$$J_{rec} \propto \exp\left(\frac{qV}{k_BT}\right) \rightarrow \text{n} = 1 \tag{3}$$

Coherent Triplet formation

$$R_{coh} \approx k_{coh}n^2p^2 \approx k_{coh}\left[n_i^2 \exp\left(-\frac{E_g}{k_bT}\right)\exp\left(\frac{qV}{k_BT}\right)\right]^2 \tag{4}$$

$$J_{rec} \propto \exp\left(\frac{2qV}{k_BT}\right) \rightarrow \text{n} = ½ \tag{5}$$

In all Rate equations, it was assumed that product of the charge carrier densities, *np* , is large compared to intrinsic charge carrier density $np >> n_i^2$, with the intrinsic charge carrier density and the non-equilibrium charge carrier densities approximated by:

$$n_i = \sqrt{N_C N_V} \exp\left(-\frac{E_g}{2k_BT}\right); \tag{6}$$

$$n = n_i \exp\left(\frac{E_{F,e} - E_C}{k_BT}\right); \tag{7}$$

$$p = n_i \exp\left(\frac{E_V - E_{F,h}}{k_BT}\right) \tag{8}$$

Supplementary Note 3: Built-in Potential

Shown in **Supplementary Figure 4** are the schematics of an intrinsic homojunction with charge carrier injection directly into the transport levels, this corresponds to the common point of view for organic electronics, a pn-homojunction and a pn-heterojunction.

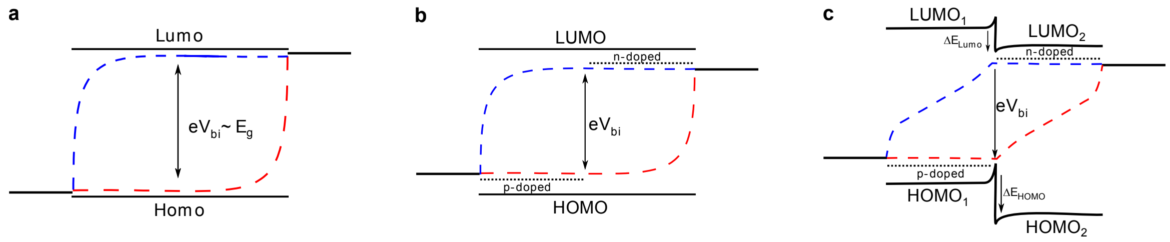

Supplementary Figure 4 – Flatband band structure

Band structure under flatband conditions: a) intrinsic homojunction with charge carrier injection into the transport levels; b) pn-homojunction, c) pn-heterojunction (different bandgaps, but smaller bandgap material lies not within the bigger bandgap material).

In the field of organic electronics, the built-in potential (built-in voltage V_{bi}) is often viewed as difference between the transport levels and as such approximately the bandgap of the semiconductor. This leads to the believe that the turn-on voltage in OLEDs, which is roughly the built-in voltage, must be equivalent to the bandgap of the material. However, from the point of view of a classical homojunction and its adaption to organic materials the built-in potential is smaller than the band gap as does the turn-on of the device. The built-in potential is the difference of the quasi-fermi energy levels of electrons $E_{\mathrm{F},n}$ and holes $E_{\mathrm{F},p}$ which for Boltzmann statistics and complete ionization yields:

$$eV_{bi} = \left(E_{F,n} - E_{F,p}\right) = k_{BT} \ln\left(\frac{n_0}{n_i}\right) + k_B T ln\left(\frac{p_0}{n_i}\right) \approx k_B T \ln\left(\frac{N_a N_d}{n_i^2}\right) = E_g + k_B T \ln\left(\frac{N_a N_d}{N_c N_V}\right) \quad (9)$$

Which for a dopant concentration N_d and N_a of 10^{15} cm^3 and effective density of states N_c and N_v of 10^{20} cm^3 leads to a reduction of the built-in potential of 0.57 eV at room temperature from the band-gap. It is to note that for lower doping concentrations the turn-on is further reduced

compared to high doping concentrations, however the current through the device is as well significantly reduced.

In case of a heterojunction device the built-in potential is determined by the offsets of the bands and the effective hole and electron densities for the two different materials:

$$eV_{bi} = \left(E_{F,n} - E_{F,p}\right) = k_B T \ln\left(\frac{n_{0,n}}{n_{o,n}}\right) + k_B T \ln\left(\frac{p_{0,p}}{n_{i,p}}\right), \tag{10}$$

which for complete ionization of dopants leads to

$$eV_{bi} \approx \left(\frac{E_{g,n}}{2} + k_B T \ln\left(\frac{N_{D,n}}{\sqrt{N_{C,n}N_{V,n}}}\right)\right) + \left(\frac{E_{g,p}}{2} + k_B T \ln\left(\frac{N_{A,p}}{\sqrt{N_{C,p}N_{V,p}}}\right)\right), \tag{11}$$

and expressed in terms of the band-gap of the p-doped material region and band-offsets ΔE_{LUMO} and ΔE_{HOMO} as defined by the schematic in **Supplementary Figure 4** can be written as

$$eV_{bi} \approx E_{g,p} - \frac{\Delta E_C + \Delta E_V}{2} + k_B T \ln\left(\frac{N_{D,n}\, N_{A,p}}{\sqrt{N_{C,n}N_{V,n}}\sqrt{N_{C,p}N_{V,p}}}\right) \tag{12}$$

Which for the same dopant concentration and effective density of states used in the example above would lead to a further reduction of the built-in potential by the average of the band-offsets. This illustrates that for a heterojunction OLED with HOMO and LUMO offsets on the order of 0.1eV a significant reduction in built-in potential and thus turn-on voltage is expected in a classic semiconductor model.

Supplementary Note 4: GPVDM simulations

For simplicity, we simulate the *J*(*V*)-characteristics of the reference rubrene/C_{60} device allowing bimolecular recombination, $R = k(np - n_i^2)$, from free holes and electrons only. The bimolecular recombination rate k was assumed to be of the value of Langevin recombination rate determined by the electron and hole mobilities throughout the device. It is well known that organic materials exhibit tail states reaching from the transport edge into the bandgap. These tail states often are modeled using an exponential dependency. While GPVDM includes these kind of trap states, it does not allow them to participate in charge transport. Instead traps are treated via SRH recombination. However, we believe that charge transport via hopping from tail states plays a significant role in organic electronics. To effectively simulate transport via these states, we replaced the HOMO / LUMO energies of rubrene and C_{60} with an effective energy representing the tail states. It is reasonable to assume that disorder will lead to 10^{15} cm^{-3} to 10^{16} cm^{-3} trap states that might reach up to (50 to 150) meV further into the gap. This leads us to an effective gap of 2 eV in case of the rubrene and 1.85 eV in case of C_{60}. Below is a detailed summary of all simulation parameters in the GPVDM notation.

Supplementary Table 1: Simulation parameter

	rubrene	c60
Electron trap density	0	0
Hole trap density ($m^{-3}eV^{-1}$)	0	0
Electron tail slope ($m^{-3}eV^{-1}$)	40E-3	20E-3
Hole tail slope (eV)	40E-3	20E-3
Electron mobility ($m^2V^{-1}s^{-1}$)	1E-5	1E-5
Hole mobility ($m^2V^{-1}s^{-1}$)	1E-4	1E-6
Relative permittivity	3	3
Number of traps	20	20
Free electron to Trapped electron (m^{-2})	1E-99	1E-99
Trapped electron to Free hole (m^{-2})	1E-99	1E-99
Trapped hole to Free electron (m^{-2})	1E-99	1E-99
Free hole to Trapped hole (m^{-2})	1E-99	1E-99
Effective density of free electron states (m^{-3})	1E+27	1E+27
Effective density of free hole states (m^{-3})	1E+27	1E+27
Xi	3.4	3.75
Eg	1.95	1.75
n_{free} to p_{free} Recombination rate constant	1.8E-12	1.8E-13

Supplementary Table 1 - GPVDM Parameters used for the simulations of a rubrene/C60 heterojunction

Supplementary Table 2: Additional Simulation Parameter

Charge carrier density on left contact	Charge carrier density on right contact	Series Resistance	Shunt Resistance
1e26 m^{-3}	*1e20* m^{-3}	*25Ω*	*35MΩ*

Supplementary Table 2 - Additional GPVDM Parameters used for the simulations of a rubrene/C60 heterojunction

Supplementary Note 5: A rate equation based model – Additional Simulations

We have explored the nature of the model solutions over a non-exclusive set of CT-rate parameters. We find the initial linear behavior at low currents is common to all models with c_{CT} between 0.99 and 0.1 for the value of 0.5 used on the man text, we have explored the relative importance of $k_{S,CT}$ to k_{CT} on the appearance of the TTA channel at moderate currents. While a suppression of either of the two rates with increasing BCP layer thickness can be used to describe the observed increase in order at medium currents (see **Supplementary Figure 5** and **Supplementary Figure 6**), we find that $k_{S,CT}$ must be the rate-limiting step to describe the exponentially decreasing J_{sc} observed for the OPV devices (see **Supplementary Figure 7**) .

Exponential dependence only on $k_{S,CT}$

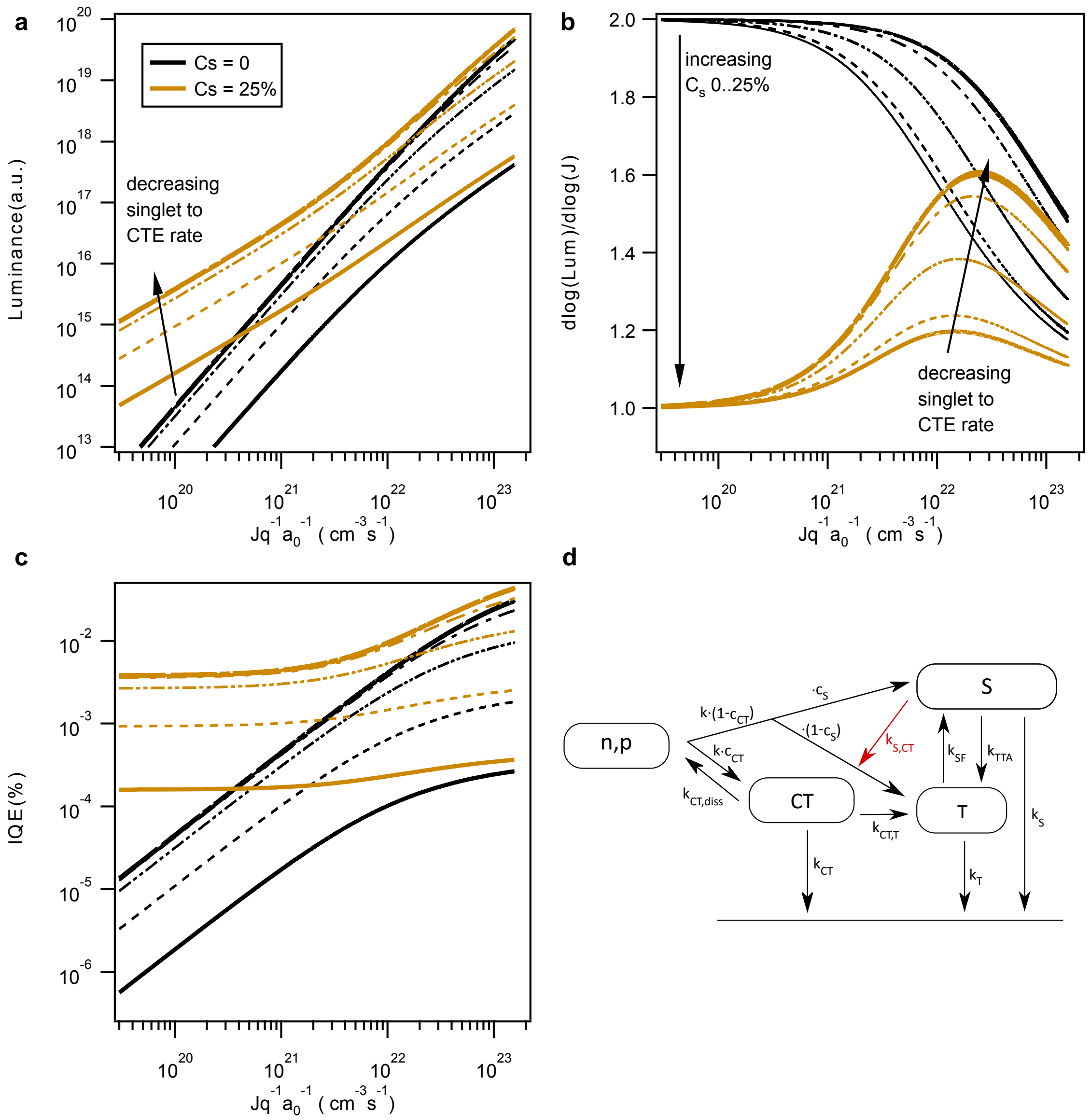

Supplementary Figure 5 - Simulated device characteristics, charge transfer state formation

a) Simulated *L*(*J*)-characteristics of rubrene based OLEDs. b) ∂log(*L*)/ ∂log(*J*), and c) current efficiency, The data was calculated using eq.(2) – eq.(4) and literature values for k_{TTA} = 10^{-14} cm^3/s, τ_T = 100 µs, τ_{SF} = 5 ps, τ_{nonRad} = 50 ps and τ_{rad} = 15 ns, c_{CT} = 50 % and $k_{T,CT}$ = 0. Assumed values: τ_{CT} = 50 ns; $\tau_{CT,T}$ = 10 ps, $\tau_{S,CT}$ = 10 ps. The fraction of directly generated singlets, C_s, has been simulated for 0 % and 25 %. To simulate the introduction of a thin BCP layer the rate $k_{S,CT}$ was multiplied by an exponential term of the form exp(-d/d_0), with d_0 = 0.5 nm and d = 0…5nm. The exponentially varied rates are highlighted in red in the schematic summary of included processes shown in d).

Exponential dependence only on $k_{CT,rec}$

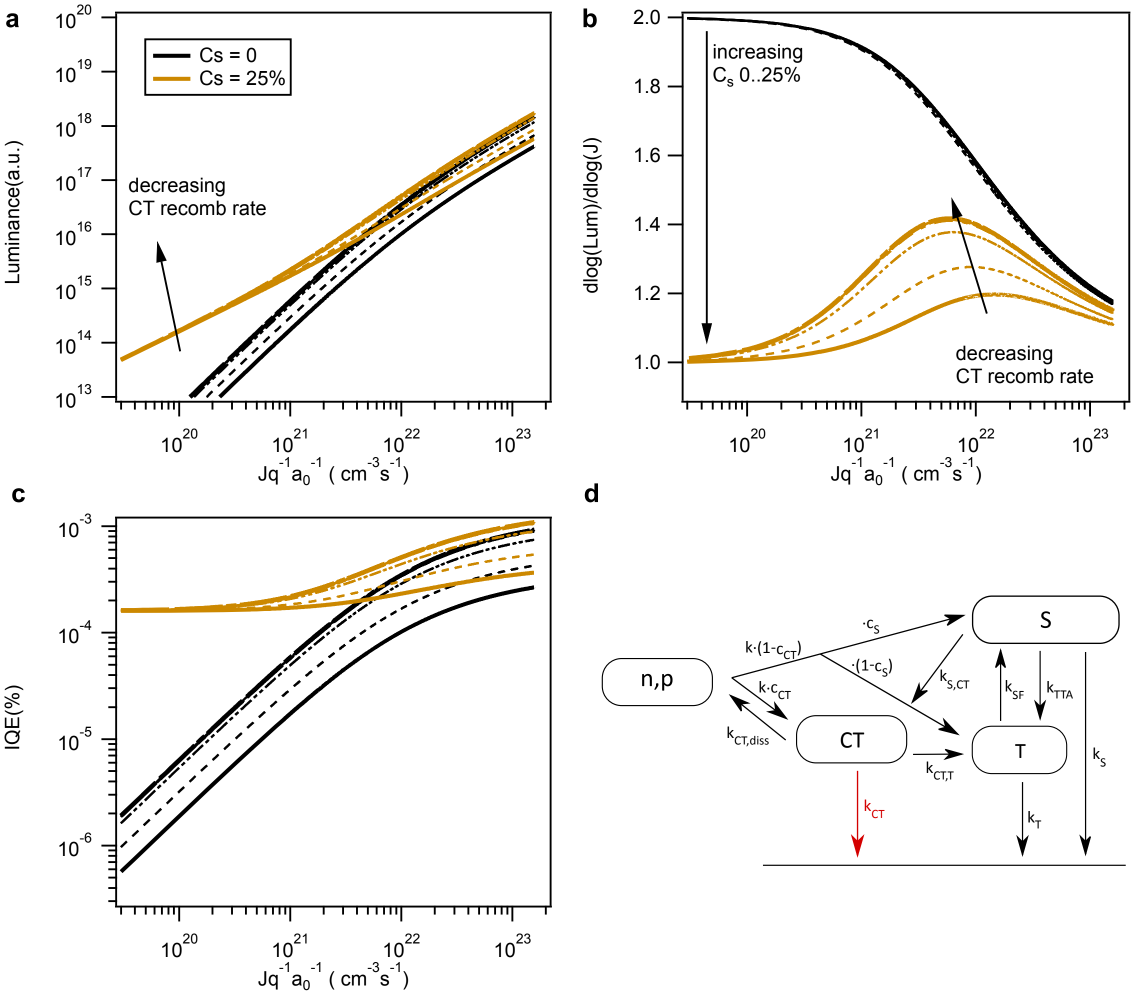

Supplementary Figure 6 – Simulated device characteristics, charge transfer state recombination

a) Simulated *L*(*J*)-characteristics of rubrene based OLEDs. b) ∂log(*L*)/ ∂log(*J*), and c) current efficiency, The data was calculated using eq.(2) – eq.(4) and literature values for k_{TTA} = 10^{-14} cm^3/s, τ_T = 100 µs, τ_{SF} = 5 ps, τ_{nonRad} = 50 ps and τ_{rad} = 15 ns, c_{CT} = 50 % and $k_{T,CT}$ = 0. Assumed values: τ_{CT} = 50 ns; $\tau_{CT,T}$ = 10 ps, $\tau_{S,CT}$ = 10 ps. The fraction of directly generated singlets, C_s, has been simulated for 0 % and 25 %. To simulate the introduction of a thin BCP layer the rate k_{CT} was multiplied by an exponential term of the form exp(-*d*/*d₀*), with d_0 = 0.5 nm and *d* = 0…5nm. The exponentially varied rates are highlighted in red in the schematic summary of included processes shown in d).

Short Circuit Current dependence

The dependence of the short circuit current on interlayer thickness was modeled using the following set of rate equations. These correspond to Eqn. (2 – 5) of the main manuscript but include a generation term of singlets on rubrene due to light absorption. Further, it was assumed that free charge carriers are extracted with yield 1.

$$\frac{J}{qa_0} + k_{CT,diss}CT = 0, \tag{13}$$

$$\frac{dCT}{dt} = -k_{CT,diss}CT - k_{CT,T}CT - k_{CT,rec}\,CT + k_{T,CT}T + k_{S,CT}S = 0, \tag{14}$$

$$\frac{dT}{dt} = k_{CT,T}CT - k_{T,CT}T - k_{T}T - k_{TTA}T^2 + 2k_{SF}S = 0, \text{ and} \tag{15}$$

$$\frac{dS}{dt} = \frac{1}{2}k_{TTA}T^2 - k_{SF}S - k_{rad}S - k_{nonRad}S - k_{S,CT}S + Generation = 0, \tag{16}$$

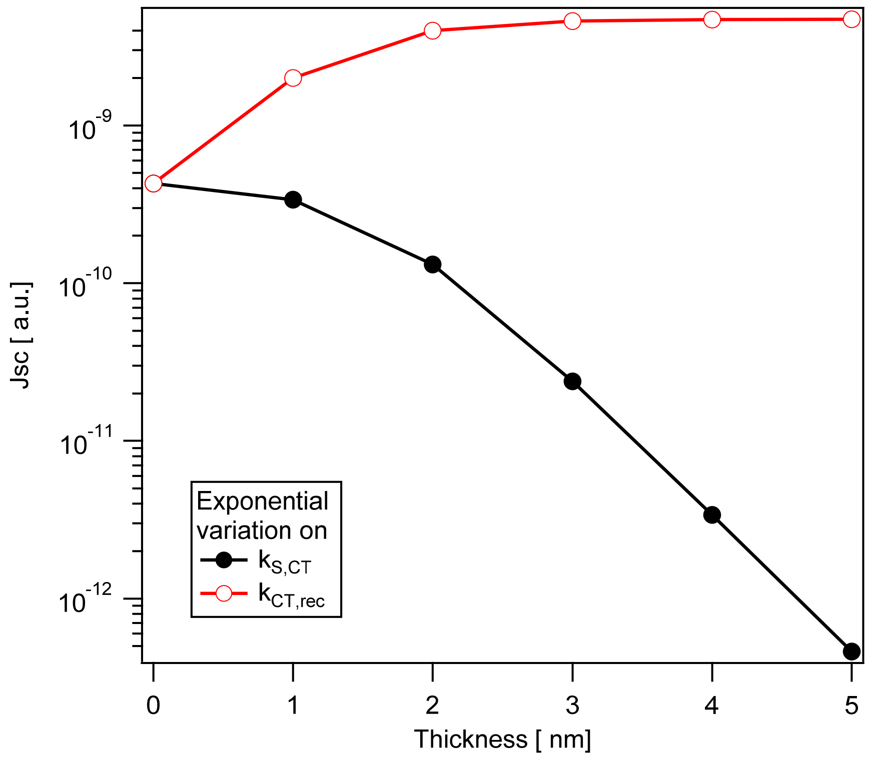

Supplementary Figure 7 - Simulated short circuit current density for various interlayer thicknesses

Simulated J_{sc} characteristics for exponential dependence on $k_{S,CT}$ and k_{CT} *Black line uses parameters identical to Fig. S6, red line identical to Fig. S7.*

Supplementary References

1 Johnson, R. C., Merrifield, R. E., Avakian, P. & Flippen, R. B. Effects of Magnetic Fields on the Mutual Annihilation of Triplet Excitons in Molecular Crystals. *Physical Review Letters* **19**, 285-287, (1967).
2 Liu, R., Zhang, Y., Lei, Y. L., Chen, P. & Xiong, Z. H. Magnetic field dependent triplet-triplet annihilation in Alq3-based organic light emitting diodes at different temperatures. *Journal of Applied Physics* **105**, 093719, (2009).
3 Lei, Y. L. *et al.* Driving current and temperature dependent magnetic-field modulated electroluminescence in Alq3-based organic light emitting diode. *Organic Electronics* **10**, 889-894, (2009).
4 Chen, Q. *et al.* Determining the Origin of Half-bandgap-voltage Electroluminescence in Bifunctional Rubrene/C60 Devices. **6**, 25331, (2016).
5 Bouchriha, H., Ern, V., Fave, J. L., Guthmann, C. & Schott, M. Magnetic field dependence of singlet exciton fission and fluorescence in crystalline tetracene at 300 K. *J. Phys. France* **39**, 257-271, (1978).
6 Smith, M. B. & Michl, J. Singlet Fission. *Chemical Reviews* **110**, 6891-6936, (2010).
